# Filtration Materials Modified with 2D Nanocomposites—A New Perspective for Point-of-Use Water Treatment

**DOI:** 10.3390/ma14010182

**Published:** 2021-01-02

**Authors:** Michał Jakubczak, Ewa Karwowska, Anita Rozmysłowska-Wojciechowska, Mateusz Petrus, Jarosław Woźniak, Joanna Mitrzak, Agnieszka M. Jastrzębska

**Affiliations:** 1 Faculty of Materials Science and Engineering, Warsaw University of Technology, Wołoska 141, 02-507 Warsaw, Poland; anita.rozmyslowska@gmail.com (A.R.-W.); mateusz.petrus.dokt@pw.edu.pl (M.P.); jaroslaw.wozniak@pw.edu.pl (J.W.); asiamitrzak@gmail.com (J.M.); agnieszka.jastrzebska@pw.edu.pl (A.M.J.); 2 Faculty of Building Services, Hydro and Environmental Engineering, Warsaw University of Technology, Nowowiejska 20, 00-653 Warsaw, Poland

**Keywords:** water treatment, filtration material, 2D nanocomposites, antibacterial properties

## Abstract

Point-of-use (POU) water treatment systems and devices play an essential role in limited access to sanitary safe water resources. The filtering materials applied in POU systems must effectively eliminate contaminants, be readily produced and stable, and avoid secondary contamination of the treated water. We report an innovative, 2D Ti_3_C_2_/Al_2_O_3_/Ag/Cu nanocomposite-modified filtration material with the application potential for POU water treatment. The material is characterized by improved filtration velocity relative to an unmodified reference material, effective elimination of microorganisms, and self-disinfecting potential, which afforded the collection of 99.6% of bacteria in the filter. The effect was obtained with nanocomposite levels as low as 1%. Surface oxidation of the modified material increased its antimicrobial efficiency. No secondary release of the nanocomposites into the filtrate was observed and confirmed the stability of the material and its suitability for practical application in water treatment.

## 1. Introduction

Low microbiological water quality remains a real problem, especially in regions with limited access to safe drinking water sources, some developing and low-income countries, and areas affected by natural disasters or terrorist attacks [1,2,3,4]. Bacteriological water contamination poses a serious threat of waterborne diseases such as diarrhea, cholera, or dysentery [5]. On-site, low cost, and easy maintenance point-of-use (POU) water treatment systems can help to deal with the problem to some extent. For effective application, POU devices must provide drinking water of sufficient microbiological quality, rapidly and independently from available water sources. These systems must be reliable, simple, and reusable [1,2,6].

Recently, many approaches have targeted the use of nanomaterials for water treatment. They cover modifications of existing water treatment methods including filtration or membrane processes [7,8,9,10]. Some promising attempts to apply the nanocomposite-based materials suitable for the POU water treatment exist. Jain and Pradeep [11] investigated polyurethane foam with nano-silver additives. Zhang and Oyanedel-Craver [10] described ceramic filters modified with nano-silver that effectively removed of *E. coli* from water. However, the need for readily available and effective materials for POU water treatment devices remains.

Two-dimensional (2D) nanocomposite structures have attracted research efforts due to their numerous potential applications. Their structures, called MXenes, come from a parental MAX phase—a hexagonal, ternary compound that forms laminar structures, built of carbides and/or nitrides, and defined by the formula M_n+1_AX_n_, where ‘M’ is a transition metal, ‘A’ refers to elements from groups 13 and 14 on the periodic table, ‘X’ is carbon and/or nitrogen, and n = 1, 2 or 3 [12,13,14,15,16]. By selective etching, using hydrofluoric acid, for instance, the ‘A’ element is removed from the MAX phase and individual sheets with additional ligands are stacked together in book-like structures. An additional treatment such as sonication affords single sheets on a nanoscale thickness [12,15,17]. Due to their combination of metal and ceramic properties, MXenes were used as heavy metal adsorbents, dyes, phosphates, and radionuclides in water treatment technologies as the membrane filtration base and electrodes for electrochemical separation and deionization processes [18,19,20,21]. 

Some studies have reported antibacterial activity for MXenes as well as some information concerning the ability of MXenes to decrease biofilm formation. Membranes activated with MXenes helped eliminate bacterial cells from filtered water [16,22]. Surface modification with other nanoparticles such as nano-zinc, nano-titanium, nano-manganese, or nano-niobium improved the antimicrobial properties of MXenes [16,22,23,24,25,26,27]. This research project developed an innovative 2D nanocomposite-modified material with potential application in the POU water treatment. Filtration materials based on polypropylene fabrics modified with Ti_3_C_2_ MXene, aluminum oxide, nano-Ag, and nano-Cu were tested for their antimicrobial properties, “self-disinfection” abilities, filtration efficiencies, and material stability (lack of nanocomponent release into the filtrate). The influence of surface oxidation of MXene Ti_3_C_2_/Al_2_O_3_/Ag/Cu-modified polypropylene fabric on its properties was also studied.

## 2. Materials and Methods 

### 2.1. Synthesis and Characterization of Nanocomposites

The Ti_3_AlC_2_ MAX phase for MXenes was produced by powder metallurgy and Spark Plasma Sintering (SPS) and described in our previous work [28]. Titanium powder (Goodfellow, Great Britain), aluminum powder (Benda-Lutz Skawina, Skawina, Poland), and synthetic graphite powder (Sigma Aldrich, St. Louis, MO, USA) were ball milled, dried, and sieved (# = 300 µm). The powders were placed in a specially designed graphite die for pressureless synthesis (molar ratio Ti:Al:C = 3:1:1.9). The SPS synthesis parameters were as follows: temperature 1300 °C; heating rate, 250 °C min^−1^; vacuum, 5 × 10^−2^ mbar. The MAX phase was ground using an automatic mortar grinder (Retsch KM100, Retsch GmbH, Haan, Germany), sieved (# = 300 µm), and etched using a 48% (*v*/*v*) concentrated hydrofluoric acid solution (24 h at room temperature, with constant stirring, 250 rpm). During this process, Al layers were removed from the MAX structure to obtain the Ti_3_C_2_ MXene. After washing with deionized water and drying at room temperature, MXenes were delaminated. The MXenes phase was magnetically stirred with a water solution of tetramethylammonium hydroxide (TMAOH) (1 mg: 1 mL: 10 mg ratio) for 24 h at room temperature and then subjected to periodical tip sonication for 6 h (1 s working/1 sec resting) with Ar bubbling (VCX 750 ultrasonic processor; Sonics, Leicestershire, UK) and subsequent washing using serial centrifugation water changing. The final 2D Ti_3_C_2_ powder was obtained after 24 h of freeze-drying (1 mbar pressure) from the suspension. 

The delaminated 2D Ti_3_C_2_ MXene was further surface-modified with Al_2_O_3_, Ag, and Cu nanoparticles using chemical precursor reagents. The Ti_3_C_2_ portion was resuspended in 5 cm^3^ of isopropanol and treated with a mixture of other reagents (see Table 1) that included aluminum isopropoxide (C_9_H_21_O_3_Al), silver acetate (C_2_H_3_AgO_2_), and copper (II) acetate (C_4_H_6_O_4_Cu) in 15 cm^3^ of isopropanol (all reagents from Sigma-Aldrich, Poznan, Poland) and stirred for 2 days (250 rpm) in closed vessels using a magnetic mixer. The samples were opened for solvent evaporation. Powder materials such as Ti_3_C_2_/Al_2_O_3_/Ag/Cu (2 wt %), Ti_3_C_2_/Al_2_O_3_/Ag/Cu (4 wt %), and Ti_3_C_2_/Al_2_O_3_/Ag/Cu (8 wt %) were subjected to further examination. It is noted that each wt % value corresponds directly to nano-metals content that were present in a 1:1 proportion.

### 2.2. Development of the Modified Polypropylene Filter Fabric 

The nanocomposite filtration materials were elaborated based on the pristine polypropylene fabric (fiber diameter = 1 µm) made by melt blowing. The nanocomposite with the highest antibacterial activity (Ti_3_C_2_/Al_2_O_3_/Ag/Cu with 8 wt % nano-metal content) was selected for material modification. Polypropylene fabric sheets were cut into 90 mm × 150 mm fragments and placed into 50 mL of an isopropanol suspension containing the nanocomponent in a flat reaction vessel. The samples were placed in a fume hood for 2 days so the volatiles would evaporate. The nanocomposite level in the filtered material was determined by weight, and the samples were stored at 4 °C. 

A part of the MXene Ti_3_C_2_/Al_2_O_3_/Ag/Cu-modified polypropylene fabric was subjected to oxidation to obtain the nanocomposite enriched with TiO_2_ (labeled as o-Ti_3_C_2_/Al_2_O_3_/Ag/Cu). It was accomplished via material incubation for 7 days at 37 °C.

The wt % of the nanocomposite content in the modified polypropylene fabric was based on the weight difference of the fabric before and after synthesis (see Equation (1)).

(1) $$\mathrm{added}\;\mathrm{amount}\;\left(\mathrm{wt}.\%\right)=\frac{w_{f}-w_{i}}{w_{i}}\times 100$$

where *w_i_* is the weight of propylene material before synthesis, and *w_f_* is the weight of material after synthesis.

### 2.3. Characterization of the Morphology and Chemical Composition of MXene-Based Composites and Surface-Modified Polypropylene Materials

The morphology of the modified polypropylene material surface was analyzed by scanning electron microscopy (SEM). The samples were coated with a layer of carbon powder using a BAL-TEC SCD 005 sputter coater and analyzed with a Zeiss Ultra Plus (Zeiss, San Diego, CA, USA) microscope at an accelerating voltage of 2.0 kV and varying magnifications. 

Fourier transform infrared spectroscopy (FTIR; Nicolet iS5 FTIR Spectrometer; Thermo Scientific, Waltham, MA, USA) identified the organic, polymeric and inorganic components of the nanocomposite-modified polypropylene fabrics. The elemental compositions of all nanomaterials and composites were investigated using X-ray fluorescence (XRF, PI 100; Polon-Izot; Warsaw, Poland). The Supplementary Materials contains those results.

The presence of nanocomposite in polypropylene materials was determined with a PI 100 benchtop X-ray fluorescence spectrometer (XRF, Polon-Izot, Warsaw, Poland), equipped with a silicon drift detector (SSD) of 125–140 eV resolution, test tube with rhodium (Rh) anode, and a multilayer monochromator of 50 keV. The measurements were performed in an air atmosphere using a measurement time of 300 s.

### 2.4. The Evaluation of Antimicrobial Properties of Nanocomposites

Pre-selection of the nanocomposites for their antibacterial properties was accomplished by diffusion. The bacterial strains used in the test (Gram-positive: *Bacillus subtilis*, *Sarcina lutea*, *Staphylococcus aureus* and Gram-negative: *Pseudomonas putida*, *Escherichia coli*) were obtained from the private collection of the Biology Department, Faculty of Building Services, Hydro, and Environmental Engineering, Warsaw University of Technology.

Microorganisms were inoculated in a form of a line on the surface of a solid nutritive culture medium (Nutrient LAB-AGAR™, Biocorp, Warsaw, Poland). The nanopowder samples were placed on the bacterial growth surface, and their cultures were incubated for 48 h at 26 °C (*Bacillus subtilis*, *Sarcina lutea,* and *Pseudomonas putida*) or 37 °C (*Escherichia coli* and *Staphylococcus aureus*). After incubation, the cultures were photographed, and the growth inhibition zones around the samples were measured. Ten measurements were taken for each nanocomponent, and the average with standard deviation was reported.

The dilution test was accomplished using two bacterial strains: *Escherichia coli* and *Staphylococcus aureus*. The experiment was carried out in a nutrient broth medium (Biocorp), diluted 1:1 with the nanocomposite suspension in a dilution ratio of q = 2, and nanocomposite concentrations from 0–500 mg L^−1^. After incubation (37 °C, 48 h), the optical densities of the cultures were measured in a MARCEL spectrophotometer at a wavelength of 610 nm, and the bacterial growth inhibition percentages were evaluated.

### 2.5. Filtration Tests

Filtration tests were carried out by applying three variants of the filtration materials:

- Pristine (unmodified) polypropylene fabrics,
- Polypropylene fabrics modified with Ti_3_C_2_/Al_2_O_3_/Ag/Cu (8 wt %),
- Polypropylene filter subjected to surface oxidation after nanocomposite enrichment with TiO_2_ crystals (labeled as o-Ti_3_C_2_/Al_2_O_3_/Ag/Cu).

The 30 mm × 90 mm filter material sheets were rolled and slid into filter columns (200 mm × 10 mm) with a uniform bed compression in all variants. The filtration beds (height, 50 mm; diameter, 10 mm), were rinsed with ≈50 cm^3^ of sterile tap water, and the dense bacterial suspension (1.3 × 10^7^ CFU ml^−1^) in dechlorinated tap water was gradually dosed with an automatic pipette. *Escherichia coli* and *Staphylococcus aureus* strains were used to approximate microbiological water contamination. The process was conducted for 150 min. Filtrate samples were collected every 30 min, and the filtration speed was periodically measured. 

The zeta electrokinetic potential (ζ) of the filtrates was analyzed with a Zetasizer Nano ZS (Malvern Instruments, Malvern, UK), equipped with an MPT-2 automatic titrator and a titration media degasser, using standard operating procedures. Simultaneously, the hydrodynamic size of the nanoparticles and the intensity of agglomerate formation in the filtrate samples were determined using dynamic light scattering (DLS).

Pearson’s linear correlation coefficient was applied to analyze the relationship between the number of bacterial cells and the zeta potential values in the filtrate. A non-parametric coefficient significance test assessed the significance of the correlation between the data. 

UV-Vis absorption of the filtrates was studied using an Evolution 210 UV-Vis spectrometer (Thermo Scientific, Waltham, MA, USA). The number of bacteria in the filtrates was determined with a pour plate technique, on Nutrient LAB-AGAR™ (Biocorp), after 48 h incubation at 37 °C, and presented as the number of colony-forming units in 1 mL of the filtrate (CFU/mL).

The “self-disinfection” properties of the filtering materials were analyzed based on microbial ability to survive in the fabric after filtration. The bacteria concentration in the fabrics was determined immediately upon filtering and after 24 h of bacterial contact with the modified material. The 3 cm^2^ fabric samples were cut using sterile scissors and washed thoroughly by shaking in a nutrient broth medium (Biocorp). The number of bacteria was determined by the pour plate method after incubation for 48 h at 37 °C. The results were given as the number of colony-forming units per cm^2^ of fabric. The efficiency of the elimination of bacteria was calculated and compared to the pristine polypropylene material.

## 3. Results

**Figure 1:**
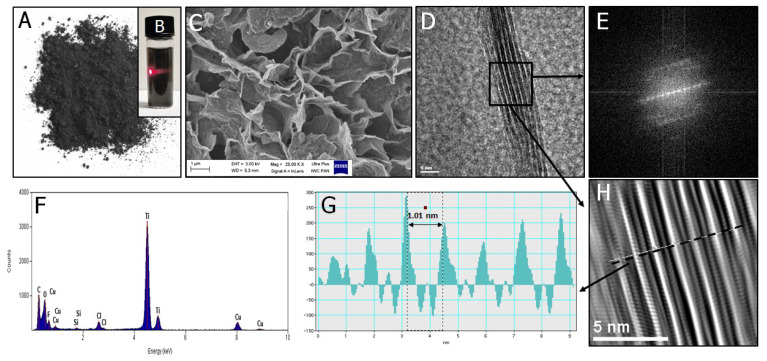
Characterization of the starting 2D Ti_3_C_2_ MXene flakes comprising (**A**) photograph of the 2D material after freeze-drying; (**B**) the observed Tyndall effect after redispersion in isopropyl alcohol; (**C**) SEM image; (**D**) high-resolution transmission electron microscopy (HRTEM) image of the edge-viewed 2D flake together with corresponding; (**E**) fast Fourier transform (FFT) image; (**F**) results of the energy dispersive spectroscopy (EDS) analysis; (**G**) intensity pattern; and (**H**) inverse fast Fourier Transfor (IFFT) image.

The present work examined the antibacterial activity of nanocomposite structures upon the addition of Ti_3_C_2_ MXene. We initially performed the analysis using the starting MXene nanoflakes, as its morphology, shape, and size influence the nanocomposite bioactivity. Characterization of unmodified, pristine Ti_3_C_2_ MXene is shown in Figure 1. Their structure and morphology were characterized using SEM, and the flakes had irregular shapes and sharp edges after freeze-drying. When redispersed in isopropyl alcohol, they formed a stable nanocolloidal solution that showed a Tyndall effect. Its presence indicated the formation of a homogeneous nanoflake nanodispersion. The high-resolution transmission electron microscopy (HRTEM) taken for a randomly chosen sheet of Ti_3_C_2_ MXene revealed its characteristic multilayered 2D structure and agreed with works reported by Naguib et al. [13], Jastrzębska et al. [25], and Rozmysłowska-Wojciechowska et al. [27]. The layered pattern was confirmed by fast Fourier Transform (FFT) and inverse fast Fourier Transform (IFFT) imaging, in which alternating layers of light and dark bands correspond to the stacking of several single Ti_3_C_2_ monolayers together with increased spacing between them. Energy dispersive spectroscopy (EDS) analysis additionally confirmed the presence of Ti and C predominantly in investigated material. The distance between maximal intensities for two adjacent Ti_3_C_2_ monolayers was 1.01 nm and agreed with our previous results [25]. SEM analyses of the 2D materials confirmed the nanometric sizes of the nanocomposites, preservation of the MXene single sheet structures, as well as the presence of nano-metallic components (see Figure S1). The nanocomponent content in the propylene material was 1.15 ± 0.2 wt %.

**Figure 2:**
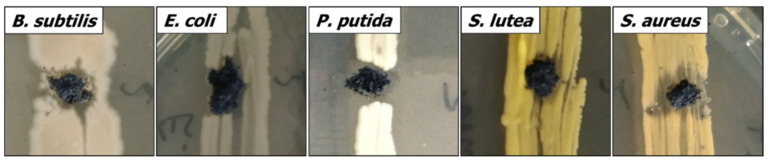
An example of the antibacterial effect of Ti_3_C_2_/Al_2_O_3_/Ag/Cu (8 wt %) in a diffusion test.

Preliminary experiments based on diffusion tests (see Figure 2 and Table 2, for details, check Tables S1 and S4–S8) revealed that all nanocomposites inhibited bacterial growth to some extent. The antibacterial activity increased with the increasing content of nanometals in the composite and depended on the type of bacteria used in the test. 

Dilution tests with *Escherichia coli* and *Staphylococcus aureus* confirmed the relationship between the nano-metal content and the intensity of microbial growth inhibition. The antibacterial activity was higher toward Gram-positive bacteria (*Staphylococcus aureus*), while results obtained for Gram-negative *Escherichia coli* were not as obvious and likely related to the protecting effect of the outer membrane of Gram-negative bacteria. For both test bacteria, inhibiting effects of the nanocomposite with 8 wt % noble metals started from 3.91 mg L^−1^, while for 4 wt %, it was 15.63 mg L^−1^. In the case of a 2 wt % sample, the inhibiting effect started from 250 mg L^−1^. The maximum inhibition values were 27.84% for *E. coli* and 34.65% for *S. aureus*. Increasing the nanocomposite concentration led to nanoparticle agglomeration and limited the antibacterial effect (the highest inhibition values were observed for the nanocomposite concentrations 31.25–62.5 mg L^−1^).

### 3.1. Structure and Properties of Modified Polypropylene Materials

**Figure 3:**
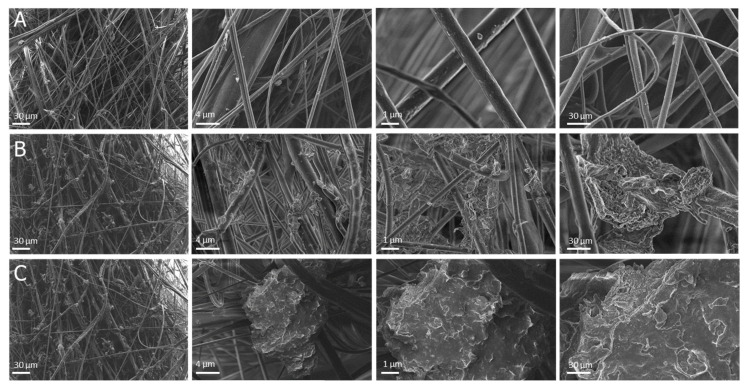
SEM images of the polypropylene filtration materials: nonmodified reference sample (**A**), and material obtained by the surface modification with Ti_3_C_2_/Al_2_O_3_/Ag/Cu (**B**), Ti_3_C_2_/Al_2_O_3_/Ag/Cu after oxidation to Ti_3_C_2_/TiO_2_/Al_2_O_3_/Ag/Cu (o-Ti_3_C_2_/Al_2_O_3_/Ag/Cu) (**C**).

Based on the above results, the Ti_3_C_2_/Al_2_O_3_/Ag/Cu containing 8 wt % Ag and Cu nanoparticles (see Figure 1C) was selected for polypropylene modification. XRF results revealed that Ti_3_C_2_/Al_2_O_3_/Ag/Cu nanostructures successfully covered the polypropylene fibers (SI; see Figures S2–S4). High-magnification SEM images showed that the polypropylene fibers were joined by the agglomerates formed by the introduced nanocomposites. The individual sheets of Ti_3_C_2_ MXene, covered with the other applied nanocomponents, were visible in the material modified with Ti_3_C_2_/Al_2_O_3_/Ag/Cu nanocomposite (see Figure 3).

The oxidation process changed the morphology and structure of polypropylene fabrics modified with the Ti_3_C_2_/Al_2_O_3_/Ag/Cu nanocomposite. In the non-oxidized material, the surface of the nanocomposite agglomerates was rather smooth (Figure 3B), while in the oxidized material, it appeared more waved and sharp (see Figure 3C). High-magnification SEM images showed that the oxidation process also resulted in diminishing the size of the nanocomposite agglomerates in the material.

FTIR analysis of the pristine polypropylene fabric used as a base for nanocomposite modifications showed peaks corresponding to C-C, C-H, CH_2_, and CH_3_ bonds. Stretching vibrations at 808, 972, and 997 cm^−1^ may be due to C-C stretches, while rocking and wagging ones occur at 841 and 1166 cm^−1^. Asymmetrical stretching vibrations at 2916 cm^−1^ correspond to CH_2_ as well as bands indicating the presence of CH_3_ groups (rocking vibrations at 841, 997, and 1166 cm^−1^, symmetrical bending at 1375 and 1453 cm^−1^, stretching at 2865 cm^−1^, and asymmetrical stretching at 2948 cm^−1^) were also detected.

FTIR peaks of fabrics modified with Ti_3_C_2_/Al_3_O_3_/Ag/Cu nanocomposite changed slightly with additional peaks at 1573 cm^−1^ and more intense ones at 1375–3342 cm^−1^. The symmetrical band detected at 1573 cm^−1^ may reflect the formation of C-O and C=O bonds, while additional bands between 500 and 1000 cm^−1^ that correspond to the Ti-O bond were not present. 

Analysis of the o-Ti_3_C_2_/Al_2_O_3_/Ag/Cu-modified fabric FTIR spectra (after oxidation) revealed some changes. The bands at 809, 840, 943, 978, and 997 cm^−1^ were slightly higher, while the ones at 1376, 1453, 2837, 2866, 2916, and 2949 cm^−1^ were significantly lower, which reflects a decrease in the number of C-C and C-H functional groups, to which forming TiO_2_ bonds, a process that consumes oxygen atoms, contributed.

### 3.2. Filtration Process 

**Figure 4:**
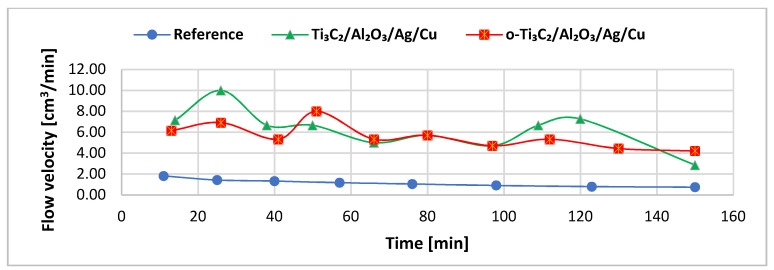
Flow velocity of the nanocomposite filtration materials tested.

The filtration process was carried out in three variants: unmodified polypropylene and filters modified with Ti_3_C_2_/Al_2_O_3_/Ag/Cu (8 wt %) and o-Ti_3_C_2_/Al_2_O_3_/Ag/Cu (8 wt %). Nanocomposite filter materials were characterized by a significantly higher flow velocity compared to the unmodified polypropylene fabric (see Figure 4). The average flow velocities were material modified with Ti_3_C_2_/Al_2_O_3_/Ag/Cu—6.27 ± 3.57 cm^3^ min^−1^, the oxidized material modified with the nanocomposite—5.62 ± 1.91 cm^3^ min^−1^, and reference polypropylene filter—1.16 ± 0.54 cm^3^ min^−1^. Initially, the flow velocity of the modified filters appeared less stable compared to the pristine polypropylene, but after 60 min, it stabilized in the o-Ti_3_C_2_/Al_2_O_3_/Ag/Cu-modified filter. 

Modification of the filter fabrics by nanocomposite incorporation did not significantly influence the bacteria removal efficiency from the filtered suspension—details are presented in Supplementary Materials (Tables S2 and S3)—exceeding 90% (for the initial concentration of bacteria in filtered water over 10^7^ CFU mL^−1^). It should be stressed that for the tested nanocomposite-based materials, this efficiency was achieved with filtration velocities three to four times higher compared to the reference material. For the oxidized Ti_3_C_2_/Al_2_O_3_/Ag/Cu-modified material, the curve describing the changes in the process efficiency was very similar to the reference filter after 1 h, while at the beginning of the process, it was approximately 30% higher. 

**Figure 5:**
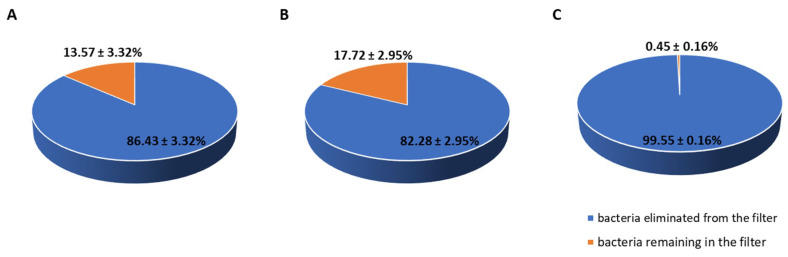
The survival of bacteria accumulated in the filter after the 24 h storage at room temperature: nonmodified polypropylene (**A**), with Ti_3_C_2_/Al_2_O_3_/Ag/Cu (**B**), with Ti_3_C_2_/Al_2_O_3_/Ag/Cu after oxidation to Ti_3_C_2_/TiO_2_/Al_2_O_3_/Ag/Cu (o-Ti_3_C_2_/Al_2_O_3_/Ag/Cu) (**C**) (one trial, four samples per trial).

The potential “self-disinfecting” properties were evaluated based on the bacteria content in filters immediately after filtration and after 24 h of storage at room temperature (22–24 °C). Samples were stored wet, in the filtration system, and exposed to light; therefore, the gradual evaporation of water that occurred must be considered. The results revealed the large antimicrobial potential of the oxidized material modified with Ti_3_C_2_/Al_2_O_3_/Ag/Cu, which eliminated 99.6% of all bacteria present in the fabric (see Figure 5; for details, check Table S9). This was not observed for the unoxidized nanocomposite material.

### 3.3. Filtrate Parameters

**Figure 6:**
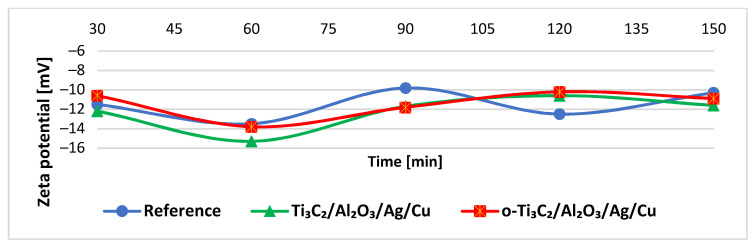
Zeta potential of the filtrates collected from the tested filtration materials.

For all filtrates, the zeta potential was negative and varied with time, from ≈−16 to −10 mV, most intensely for the filtrate from the unmodified material (see Figure 6). It is worth noting the zeta potential of the filtrate samples collected from the nanocomposite-modified filters approximated those obtained from the reference material; this indicated that the nanocomponents were not flushed out intensively from the material during filtration. 

**Figure 7:**
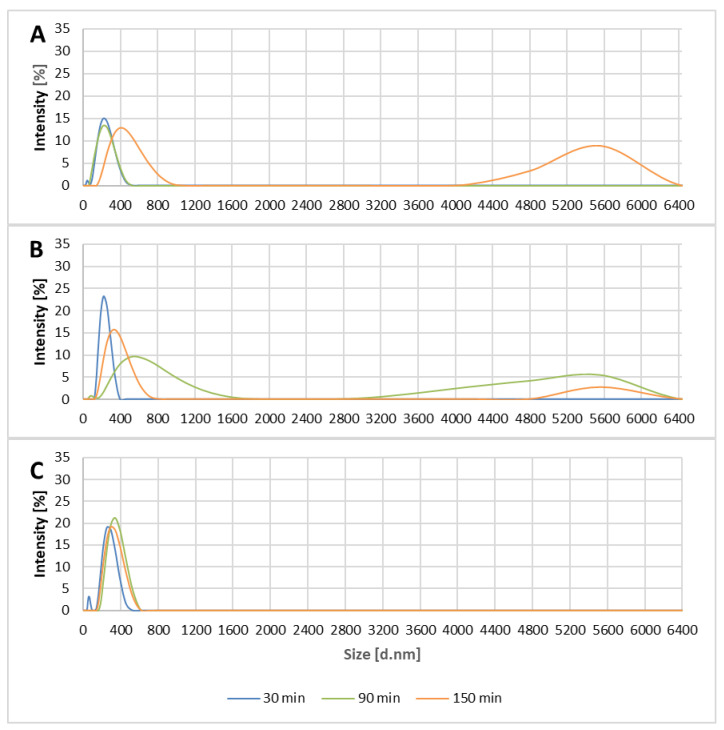
The agglomerate appearance intensity in the collected filtrates: reference polypropylene material (**A**), Ti_3_C_2_/Al_2_O_3_/Ag/Cu-modified (**B**), and o-Ti_3_C_2_/Al_2_O_3_/Ag/Cu-modified (**C**).

The particle size distribution was similar in filtrates for both reference and modified filters. We assumed that the particles present in the filtrates were related to the pristine polypropylene matrix or the bacterial cell fragments passing through the filter. The signals detected might be also due to the presence of the nano-sized particles in tap water. Furthermore, smaller agglomerates (below 1000 nm) that appeared in all filtrates and were observed both in the tested and the reference material may be due to the presence of nanoparticles in the used test suspension. The second group of agglomerates (over 2800 nm) can be due to secondary contamination of the filtrate with the material collected on the filters. However, it should be stressed that the group of the larger clusters was not detected in filtrates collected from the nanocomposite filtration material modified with oxidized Ti_3_C_2_ MXene; this suggested a limited influence of the material on biological material leakage from the filter (see Figure 7). This effect was not observed in the unoxidized material.

### 3.4. UV-Vis Study of The Filtrate

**Figure 8:**
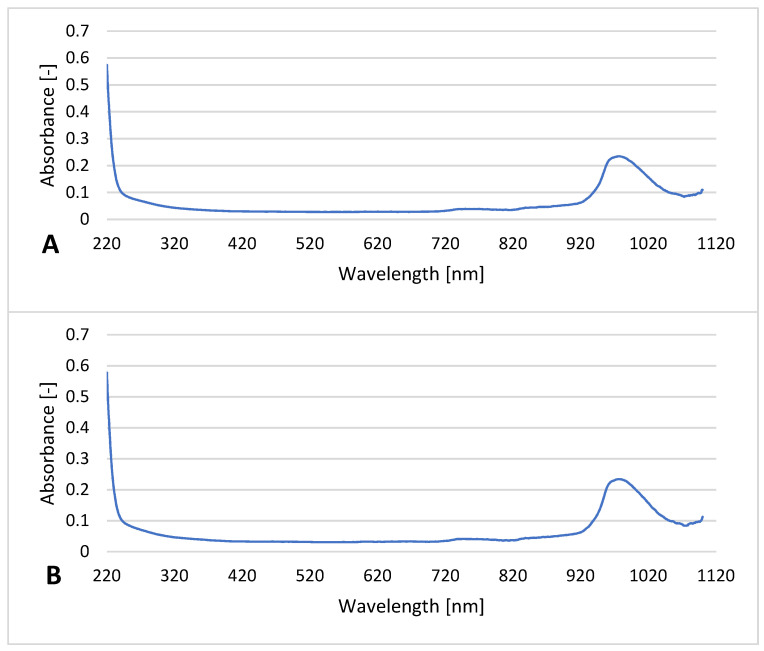
UV-Vis spectra of the filtrates from unmodified polypropylene (**A**) and material modified with o-Ti_3_C_2_/Al_2_O_3_/Ag/Cu (**B**).

UV-Vis analysis of the filtrates revealed similar spectra for all filtrates with one significant peak, with a λ_max_ (~0.24) at ≈970 nm and some slight fluctuations in the region between 720–820 nm (absorbances < 0.05; see Figure 8). The extremely consistent spectra confirmed that the nanocomponents introduced remained in the filters. The peak observed in all filtrates (including the reference) between 940 and 1040 nm may be related to the presence of bacterial cell fragments or bacterial suspension compounds. 

## 4. Discussion

The nanomaterials characterized by a specific surface offers the potential for their functionalization with a variety of particles and structures, which confers the base material with new and desirable properties. We focused on the application of 2D structure-based composites to create a novel filtering material for “point-of-use” water treatment systems.

The first milestone was the synthesis of the series of innovative nanoproducts composed of elements with potential antimicrobial activity. Their synthesis should be relatively straightforward based on commercially available reagents, and their combination should reveal their antimicrobial impacts. 

For nanocomposite construction, MXenes were selected due to the possibility of precise pore size control and hydrophilic properties that allow MXenes to contribute to liquid transport efficiency and ion release, fouling reduction, and biofilm formation. All attributes result in longer-lived filtration units [20,24,26,29]. The applied noble metal nano-metals had been used previously as antibacterial agents in different applications [30]. In addition, the antimicrobial properties of Al_2_O_3_ had been observed in several studies [31,32,33].

Diffusion and dilution tests confirmed that the novel nanocomponents limited bacterial growth. The nanocomposites with 8 wt % of metallic nanoparticles were the most effective toward bacteria, especially Gram-positive strains. An increase in the antibacterial effect related to the nanometal amount suggested that the diffusion of Ag and Cu ions from the nanocomposite was the main mechanism of the antimicrobial action of the powdery nanocomponents, while Ti_3_C_2_ MXene and Al_2_O_3_ nanoparticles played minor roles. The action of nano-Ag and nano-Cu is related to the adhesion of nanoparticles to the cell membrane and following interactions with its components resulting in changes of the cell permeability and affecting cellular respiration, enzymatic, gene expression, and metabolic processes [34,35,36,37]. The induction of oxidative stress associated with the generation of reactive oxygen species (ROS) and silver ion release should also be considered [38,39,40,41]. On the other hand, the tendency of Ti_3_C_2_ MXenes to agglomerate [42] due to strong van der Waals interactions helps keep the metal nanoparticles inside the agglomerates, which decreases their diffusion potential and impact on bacterial cells.

The production of the POU filtration material needs a simple, available base suitable for effective modifications. The synthetic fibers serve both as a base material and an effective nanocomponent carrier. The impregnation of polyester fibers with copper ions (3–10 wt %) produced the active antibacterial, anti-fungal, and anti-virus material [43]. The antibacterial effect was observed for nano-Ag-modified nylon and polyester [44,45], polypropylene [46], and polyamide 6™ [47].

In this research, the polypropylene base was used for nanocomposite modifications. The synthetic procedure was not complicated and very effective at producing the desired material. The modification did not change the chemical structure of the carrier fiber, which remained typical for the pristine material [48]. FTIR spectra showed that peak assignments typical for the pure polypropylene also occurred in the modified material; however, additional C-O and C=O bonds confirmed the appearance of Ti_3_C_2_ MXene. Successful modification of the polypropylene fabrics was also confirmed by SEM analysis and revealed the nanostructure presence. The visible changes that resulted from the oxidation of Ti_3_C_2_/Al_2_O_3_/Ag/Cu nanocomposite toward Ti_3_C_2_/TiO_2_/Al_2_O_3_/Ag/Cu on the surface of the polypropylene material resulted in the formation of anatase crystals (TiO_2_).

Our studies showed that the modified polypropylene fabrics can create a non-compressible, solid bed. Moreover, the modifications changed the structure of the material and caused a noticeable increase in the flow velocity. This astonishing effect brings an essential advantage for the material, solving one of the problems for point-of-use devices [49].

The effective “transfer” of antibacterial activity from the powder nanocomposite to the modified polypropylene material was another accomplishment of this research. The nanocomposite with the highest levels of metallic nanoparticles (8 wt %) was applied in the filtration experiments. In contrast to tests with powdery nanocomposites, the presence of Ti_3_C_2_/Al_2_O_3_/Ag/Cu did not improve the filtration efficiency of the bacterial suspension (artificially “contaminated water”). For filter fabrics modified with oxidized Ti_3_C_2_/Al_2_O_3_/Ag/Cu, a slight improvement in bacteria removal was observed compared to the pristine polypropylene fabric, especially at the beginning of the process. However, the material with the oxidized Ti_3_C_2_ MXene nanocomposite worked effectively over the entire experiment with superior antimicrobial activity that diminished bacterial numbers by two orders of magnitude.

Recently, the self-cleaning properties of filtration membranes and filters have been investigated. A majority of these works focused on “in situ” decomposition of dyes and chemicals. However, the aspect of killing residual surface bacteria was marginalized. The self-cleaning activity of BC-SiO_2_-TiO_2_ (where BC stands for bacterial cellulose) photocatalytic membranes enhanced by Ag doping was studied by Rahman et al. [50]. They reported a 41% efficiency increase of crystal violet dye decomposition relative to the reference sample. Furthermore, the samples showed some antibacterial activity toward *Bacillus subtilis*, *Klebsiella pneumoniae*, *salmonella arizonae*, *E. coli,* and *Kluyvera spp.* strains caused by the presence of nanosilver [50]. Self-cleaning MIL-125(Ti)/PVDF (polyvinylidene fluoride) hybrid membranes were obtained by Zhou et al. [51], who also tested their activity in contact with dyes and bacteria cells. Selected samples showed extraordinary efficiency (up to 100%, depending on the Ti content) during three filtration cycles and antibacterial properties against *E. coli* bacteria [51]. Ma et al. [52] investigated the efficiency of oil/water separation with nanofibrous membranes, which was characterized by remarkable recyclability, antibacterial properties, as well as environmentally friendly and low-cost features [52]. Li et al. [53] observed the ability of graphitic carbon nitride (g-C_3_N_4_) nanosheets to form self-cleaning membranes with antibacterial activity (≈100%). However, after several cycles, defects appeared that resulted in higher membrane permeability [53]. The self-disinfection properties were also tested by Lei et al. [54]. Filters were designed based on aminopyridine conjugated microporous polymer nanotubes to capture the PM 2.5 dust fraction. The antibacterial activity against *E. coli* enabled the rapid neutralization of tested bacteria and killed residual bacteria. This prevented secondary contamination of the filtrate. On the other hand, frequently used filtration systems differ significantly from the one presented here, which can be applied at places that lack sanitation [54]. Stan et al. [55] removed residual bacteria of the cotton fabrics by coating them with graphene oxide/TiO_2_. After 2 h contact time, the growth of *S. aureus* was inhibited slightly, although *E. faecalis* was not affected. Fortunately, after 24 h, both bacterial strains were reduced significantly. It is worth noting that these properties were achieved under static conditions, and their performance in dynamic filtration systems has not been studied [55].

There is evidence that the antimicrobial activity of nanoparticle-modified membranes makes them less sensitive to biological fouling [56]. This research obtained a material with “self-disinfecting” properties, which should help with repeated use of the filter-equipped devices. After 24 h, the number of bacteria dropped by 99.6 wt % in a novel filter modified with the TiO_2_ enriched nanocomposite. The formation of titanium dioxide (anatase crystals) on the surface of Ti_3_C_2_, acting as a robust antibacterial agent, was reported by Rasool et al. [16] and suspected to be the predominant factor of the antibacterial properties of Ti_3_C_2_ MXene. Antimicrobial properties of titanium dioxide have been reported in several works [29,57], but its activity was strongly related to UV irradiation [58]. In this research, antibacterial effects were readily visible despite the absence of UV stimulation.

The most essential quality for the practical usage of the filtration material is to procure a safe, high-quality filtrate. The effective elimination of microbiological contaminants must be accompanied by material stability, which is critical especially for nanocomponent-modified filters, because it avoids secondary contamination of the filtrate with nanoparticles.

The measurement of the electrochemical (zeta) potential of the filtrates helped evaluate the electrostatic interactions between the solution and the particles (e.g., bacteria cells), including the effects of the bacterial adsorption on the surface charge of bacterial cells [59]. The bacteria applied in the filtration experiment—*E. coli* and *S. aureus*, possess a negative zeta potential in drinking water (~−40 and −17 mV, respectively) [60]. The zeta potentials measured correspond to the bacterial cells that remained in the filtrate samples. According to literature data, Ti_3_C_2_ MXene, Al_2_O_3_, Ag, and Cu nanoparticles all contribute to negative zeta potentials in water, especially at alkaline pH [27,61,62,63]. Therefore, the release of nanocomponents was readily observed and yielded information regarding the instability of the material. In our research, this was not observed; the zeta values were similar for both unmodified polypropylene and nanocomposite-modified materials. The effect was also confirmed by UV-Vis spectral analyses of the filtrates that revealed no significant differences between filtrates collected after modified filters and the unmodified reference material. A peak detected in each sample was probably related to the presence of the biological fragments. 

The DLS analysis revealed that certain levels of small particles and agglomerates were present in all filtrates. These likely included cells and their organelles [64], residues of the microbiological cultivation media, and the particles present in tap water used for the bacterial suspension preparation. However, they were also present in reference samples, so they were not related to the nanocomponents flushed from the filtration bed.

## 5. Conclusions

The filtration materials applied in point-of-use water treatment systems must meet certain basic requirements: synthetic and manufacturing ease, chemical and physical stability, and effectiveness in the elimination of microorganisms. The material presented in this work yielded additional advantages: improved filtration velocity with an undisturbed or improved elimination efficiency of microorganisms as compared to pristine polypropylene. The elaborated filtration material modified with Ti_3_C_2_/Al_2_O_3_/Ag/Cu was obtained using commercial reagents and a readily available and cheap polypropylene base. The 1 wt % content of the nanocomposite did not increase the fabric cost significantly. The filter collected almost ≈10^5^ bacterial cells per 1 cm^2^ of material, which makes it suitable for the effective removal of typical microbiological water contaminants. The best effect was achieved by oxidation of the modified material, which increased its antimicrobial impact. The self-disinfecting properties eliminated ≈99.6 wt % of bacteria collected on the filter for both *Escherichia coli* (typical for fecal contamination) and potentially pathogenic *Staphylococcus aureus*. The material is environmentally safe due to its stability and lack of nanocomposite material release. This work clearly showed that 2D MXene nanocomposites are a promising new perspective for point-of-use water treatment. 

## Figures and Tables

**Table 1:** Reagent amounts used for nanocomponent syntheses with different levels of metallic nanoparticles.

	Ti_3_C_2_/Al_2_O_3_/Ag/Cu		Al_2_O_3_/Ag/Cu			
Content of Metallic Nanoparticles	2 wt %	4 wt %	8 wt %	2 wt %	4 wt %	8 wt %
C_9_H_21_O_3_Al [mg]	20	20	20	378	356	312
C_2_H_3_AgO_2_ [mg]	10	20	40	10	20	40
C_4_H_6_O_4_Cu [mg]	12	24	48	12	24	48
Ti_3_C_2_ [mg]	378	356	312	–	–	–

**Table 2:** Growth inhibition zones (mm) in the diffusion test of nanopowders with different levels of metallic nanoparticles.

Bacteria	The Growth Inhibition Zones (mm) for Different Noble Metal Contents in Ti_3_C_2_/Al_2_O_3_/Ag/Cu Nanocomposite		
	2 wt %	4 wt %	8 wt %
*Escherichia coli*	0.70 ± 0.10	1.31 ± 0.18	2.40 ± 0.17
*Pseudomonas putida*	1.70 ± 0.10	1.93 ± 0.16	4.47 ± 0.25
*Sarcina lutea*	1.50 ± 0.14	0.29 ± 0.08	0.60 ± 0.06
*Staphylococcus aureus*	2.02 ± 0.08	1.08 ± 0.10	2.42 ± 0.09
*Bacillus subtilis*	0.69 ± 0.09	0.27 ± 0.03	2.43 ± 0.23

## Data Availability

The data presented in this study are partially available in supplementary material.

## Supplementary Materials

The following are available online at https://www.mdpi.com/1996-1944/14/1/182/s1; Table S1. The growth inhibition zones (mm) in the diffusion test of the Ti_3_C_2_ MXene and Al_2_O_3_ nanoparticles; Table S2. The number of colony-forming units (CFU) found in filtrate after certain time; Table S3. Percentage efficiency of filtration process; Table S4. Growth inhibition zones (mm) in the diffusion test of nanopowders with different levels of metallic nanoparticles, and with calculated statistics for *Bacillus subtilis*; Table S5. Growth inhibition zones (mm) in the diffusion test of nanopowders with different levels of metallic nanoparticles, and with calculated statistics for *Escherichia coli*; Table S6. Growth inhibition zones (mm) in the diffusion test of nanopowders with different levels of metallic nanoparticles, and with calculated statistics for *Pseudomonas putida*; Table S7. Growth inhibition zones (mm) in the diffusion test of nanopowders with different levels of metallic nanoparticles, and with calculated statistics for *Sarcina lutea*; Table S8. Growth inhibition zones (mm) in the diffusion test of nanopowders with different levels of metallic nanoparticles, and with calculated statistics for *Staphylococcus aureus*; Table S9. Results for “self-disinfection” properties investigation, as well as its statistical analysis; Figure S1. SEM images of the nanocomposite powders: Ti_3_C_2_/Al_2_O_3_/Ag/Cu (2 wt %) (A), Ti_3_C_2_/Al_2_O_3_/Ag/Cu (4 wt %) (B), Ti_3_C_2_/Al_2_O_3_/Ag/Cu (8 wt %) (C). The insets correspond to BSE imaging of metal particles; Figure S2. XRF spectra of reference polypropylene material; Figure S3. XRF spectra of Ti_3_C_2_/Al_2_O_3_/Ag/Cu-modified material; Figure S4. XRF spectra of o-Ti_3_C_2_/Al_2_O_3_/Ag/Cu-modified material.
