# Supplementary material for: Filtration Materials Modified with 2D Nanocomposites—A New Perspective for Point-of-Use Water Treatment
Source: Materials (Basel). 2021 Jan 2;14(1):182. doi: 10.3390/ma14010182 (PMC7795578; doi:10.3390/ma14010182)
Supplement: Supplementary file 1 [file materials-14-00182-s001.pdf]

materials

Supplementary Materials

Filtration Materials Modified with 2D Nanocomposites—A New Perspective for Point-of-Use Water Treatment

Michał Jakubczak [1,*], Ewa Karwowska [2,*], Anita Rozmysłowska-Wojciechowska [1], Mateusz Petrus [1], Jarosław Woźniak [1], Joanna Mitrzak [1] and Agnieszka M. Jastrzębska [1]

1 Faculty of Materials Science and Engineering, Warsaw University of Technology, Wołoska 141, 02-507 Warsaw, Poland; anita.rozmyslowska@gmail.com (A.R.-W.); mateusz.petrus.dokt@pw.edu.pl (M.P.); jaroslaw.wozniak@pw.edu.pl (J.W.); asiamitrzak@gmail.com (J.M.); agnieszka.jastrzebska@pw.edu.pl (A.M.J.)
2 Faculty of Building Services, Hydro and Environmental Engineering, Warsaw University of Technology, Nowowiejska 20, 00-653 Warsaw, Poland
* Correspondence: michal.jakubczak.dokt@pw.edu.pl (M.J.); ewa.karwowska@pw.edu.pl (E.K.); Tel.: +48-222-345-944 (E.K.)

Table S1. The growth inhibition zones (mm) in the diffusion test of the Ti_3C_2 MXene and Al_2O_3 nanoparticles.

Bacteria	The Growth Inhibition Zones (mm) for Negative Control Samples	
	Ti_3C_2 MXene	Al_2O_3 nanoparticles
Bacillus subtilis	0.00 ± 0.00	0.00 ± 0.00
Escherichia coli	0.00 ± 0.00	0.00 ± 0.00
Pseudomonas putida	0.00 ± 0.00	0.00 ± 0.00
Sarcina lutea	0.00 ± 0.00	0.00 ± 0.00
Staphylococcus aureus	0.00 ± 0.00	0.00 ± 0.00

Table S2. The number of colony forming units (CFU) found in filtrate after certain time.

Time [min]	[CFU/ml]		
	Reference	$Ti_3C_2/Al_2O_3/Ag/Cu$	o-$Ti_3C_2/Al_2O_3/Ag/Cu$
30	5.3×10^6	5.8×10^6	1.3×10^6
60	1.1×10^6	5.3×10^6	1.3×10^6
90	2.9×10^6	3.3×10^6	3.2×10^6
120	1.2×10^6	1.4×10^6	1.9×10^6
150	3.2×10^6	6.2×10^6	3.8×10^6

Table S3. Percentage efficiency of filtration process.

Time [min]	[%]		
	**Reference**	**$Ti_3C_2/Al_2O_3/Ag/Cu$**	**o-$Ti_3C_2/Al_2O_3/Ag/Cu$**
30	59.70	55.91	89.84
60	91.67	59.55	90.29
90	78.40	75.15	75.76
120	91.06	89.09	85.83
150	76.06	52.80	71.44

Table S4. Growth inhibition zones (mm) in the diffusion test of nanopowders with different levels of metallic nanoparticles, and with calculated statistics for *Bacillus subtilis.*

	**$Ti_3C_2/Al_2O_3/Ag/Cu$ (2%)**	**$Ti_3C_2/Al_2O_3/Ag/Cu$ (4%)**	**$Ti_3C_2/Al_2O_3/Ag/Cu$ (8%)**
Arithmetic mean	0.69	0.27	2.43
Standard deviation	0.09	0.03	0.23
Number of counts	10	10	10
Kolmogorov-Smirnov test value	0.958	1.294	0.795
K-S critical value	1.358	1.358	1.358
t-test	1.2×10^{-15}	9.9×10^{-18}	5.6×10^{-18}
t-test critical value	0.05	0.05	0.05

Table S5. Growth inhibition zones (mm) in the diffusion test of nanopowders with different levels of metallic nanoparticles, and with calculated statistics for *Escherichia coli*.

	**$Ti_3C_2/Al_2O_3/Ag/Cu$ (2%)**	**$Ti_3C_2/Al_2O_3/Ag/Cu$ (4%)**	**$Ti_3C_2/Al_2O_3/Ag/Cu$ (8%)**
Arithmetic mean	0.70	1.31	2.40
Standard deviation	0.10	0.18	0.17
Number of counts	10	10	10
Kolmogorov-Smirnov test value	0.419	0.515	0.748
K-S critical value	1.358	1.358	1.358
t-test	1.5×10^{-14}	2.9×10^{-15}	5.7×10^{-20}
t-test critical value	0.05	0.05	0.05

Table S6. Growth inhibition zones (mm) in the diffusion test of nanopowders with different levels of metallic nanoparticles, and with calculated statistics for *Pseudomonas putida*.

	**$Ti_3C_2/Al_2O_3/Ag/Cu$ (2%)**	**$Ti_3C_2/Al_2O_3/Ag/Cu$ (4%)**	**$Ti_3C_2/Al_2O_3/Ag/Cu$ (8%)**
Arithmetic mean	1.70	1.93	4.47
Standard deviation	0.10	0.16	0.25
Number of counts	10	10	10
Kolmogorov-Smirnov test value	0.546	0.733	0.807
K-S critical value	1.358	1.358	1.358
t-test	7.8×10^{-22}	4.8×10^{-19}	5.7×10^{-22}
t-test critical value	0.05	0.05	0.05

Table S7. Growth inhibition zones (mm) in the diffusion test of nanopowders with different levels of metallic nanoparticles, and with calculated statistics for *Sarcina lutea*.

	Ti_3C_2/Al_2O_3/Ag/Cu (2%)	Ti_3C_2/Al_2O_3/Ag/Cu (4%)	Ti_3C_2/Al_2O_3/Ag/Cu (8%)
Arithmetic mean	1.50	0.29	0.60
Standard deviation	0.14	0.08	0.06
Number of counts	10	10	10
Kolmogorov-Smirnov test value	0.562	0.516	0.798
K-S critical value	1.358	1.358	1.358
t-test	4.9×10^{-18}	5.7×10^{-10}	1.7×10^{-17}
t-test critical value	0.05	0.05	0.05

Table S8. Growth inhibition zones (mm) in the diffusion test of nanopowders with different levels of metallic nanoparticles, and with calculated statistics for *Staphylococcus aureus*.

	Ti_3C_2/Al_2O_3/Ag/Cu (2%)	Ti_3C_2/Al_2O_3/Ag/Cu (4%)	Ti_3C_2/Al_2O_3/Ag/Cu (8%)
Arithmetic mean	2.02	1.08	2.42
Standard deviation	0.08	0.10	0.09
Number of counts	10	10	10
Kolmogorov-Smirnov test value	0.570	0.579	0.403
K-S critical value	1.358	1.358	1.358
t-test	1.2×10^{-24}	4.1×10^{-18}	1.9×10^{-25}
t-test critical value	0.05	0.05	0.05

Table S9. Results for 'self-disinfection' properties investigation, as well as its statistical analysis.

	CFU/cm² after 0 h			CFU/cm² after 24 h			% of efficiency		
	**A**	**B**	**C**	**A**	**B**	**C**	**A**	**B**	**C**
**Arithmetic mean**	1.1×10^5	1.3×10^5	4.9×10^5	1.4×10^4	2.3×10^4	2.2×10^3	86.43	82.28	99.55
**Standard deviation**	8.7×10^3	1.4×10^4	9.4×10^4	2.3×10^3	4.9×10^3	2.8×10^2	3.32	2.95	0.16
**Number of counts**	4	4	4	4	4	4	4	4	4
**Dixon's Q-test result**	no outliers	no outliers	no outliers	no outliers	no outliers	no outliers	no outliers	no outliers	no outliers
**F-test**	-	4.6×10^1	2.7×10^3	-	2.5×10^1	5.7×10^3	-	8.5×10^1	3.9×10^4
**t-test**	-	2.3×10^2	8.9×10^5	-	1.8×10^2	2.3×10^5	-	7.7×10^4	1.2×10^5
**t-test critical value**	0.05	0.05	0.05	0.05	0.05	0.05	0.05	0.05	0.05

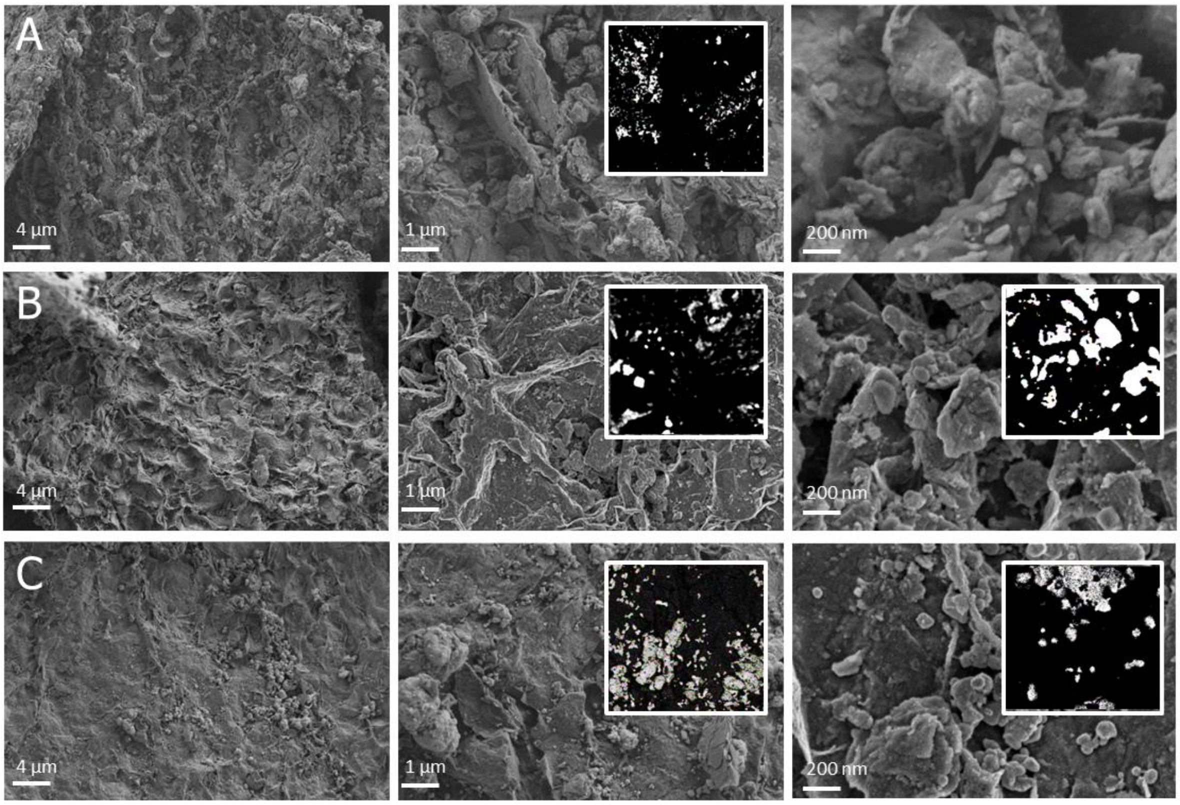

Figure S1. SEM images of the nanocomposite powders: Ti_3C_2/Al_2O_3/Ag/Cu (2 wt.%) (**A**), Ti_3C_2/Al_2O_3/Ag/Cu (4 wt.%) (**B**), Ti_3C_2/Al_2O_3/Ag/Cu (8 wt.%) (**C**). The insets correspond to BSE imaging of metal particles.

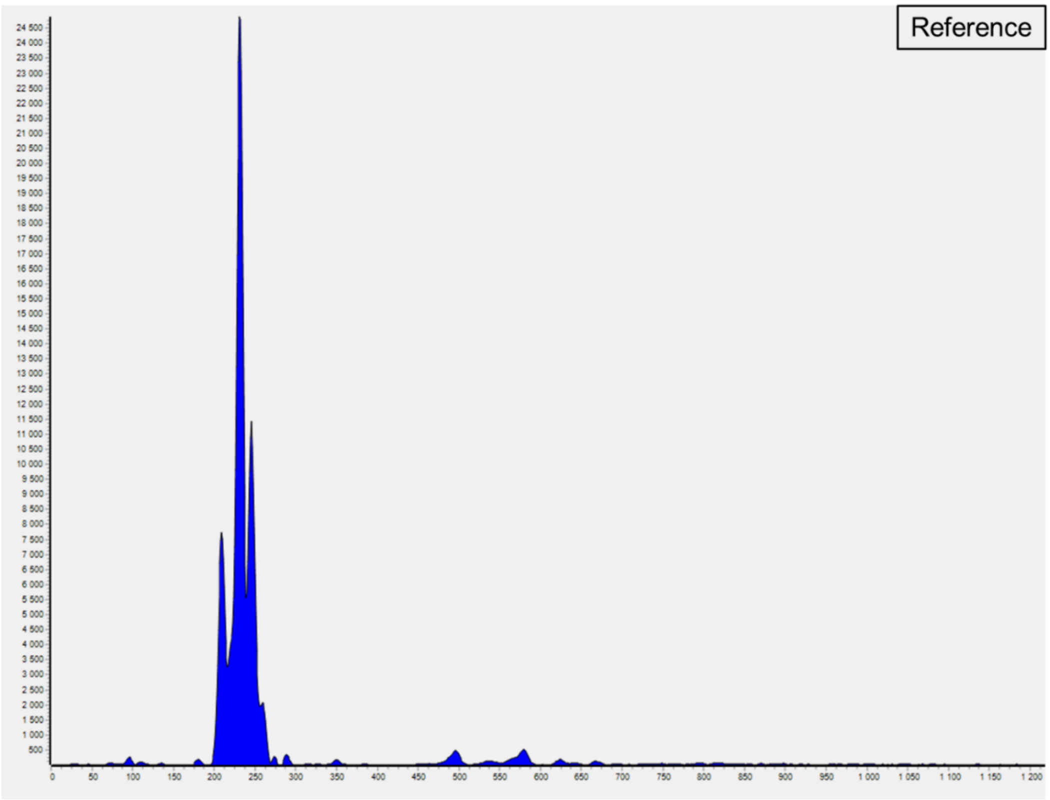

Figure S2. XRF spectra of reference polypropylene material.

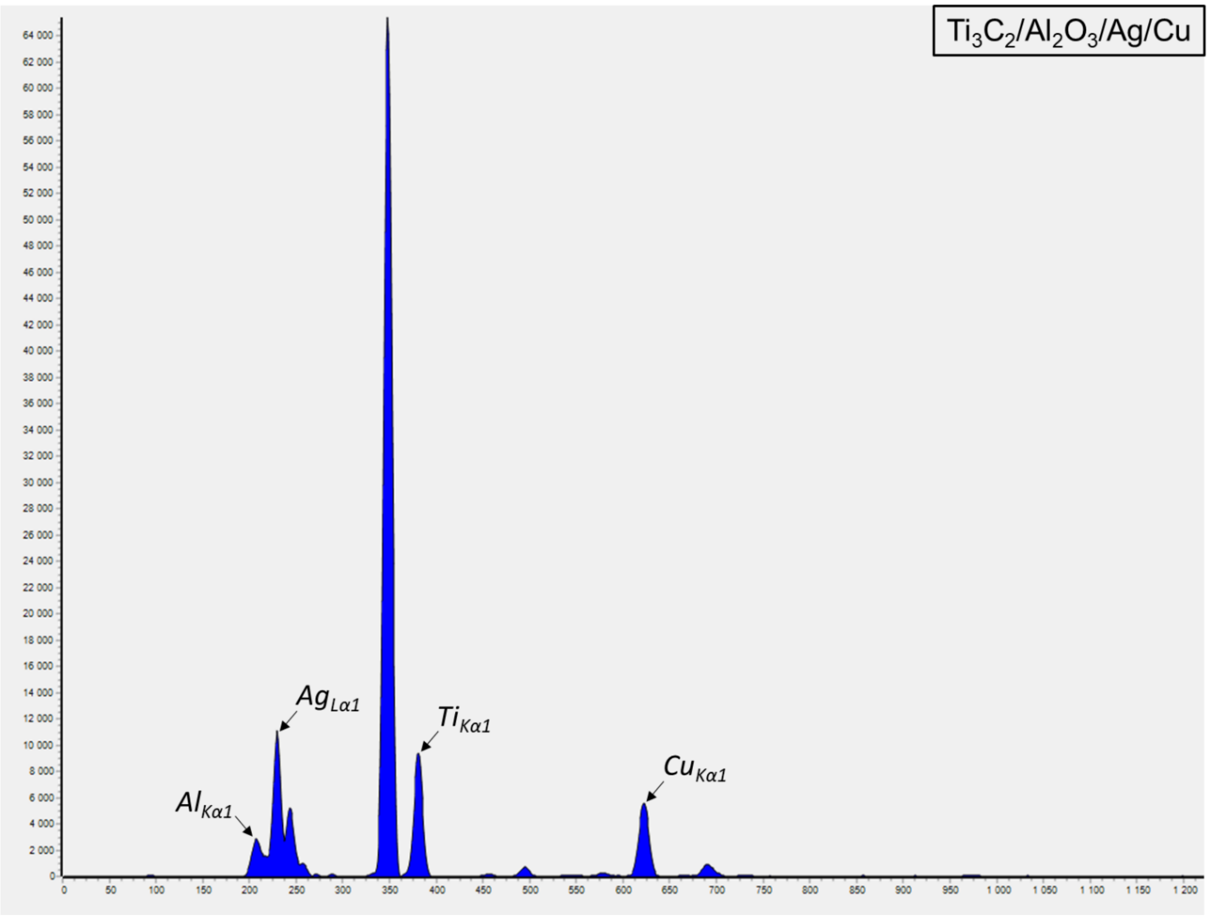

Figure S3. XRF spectra of $Ti_3C_2/Al_2O_3/Ag/Cu$-modified material.

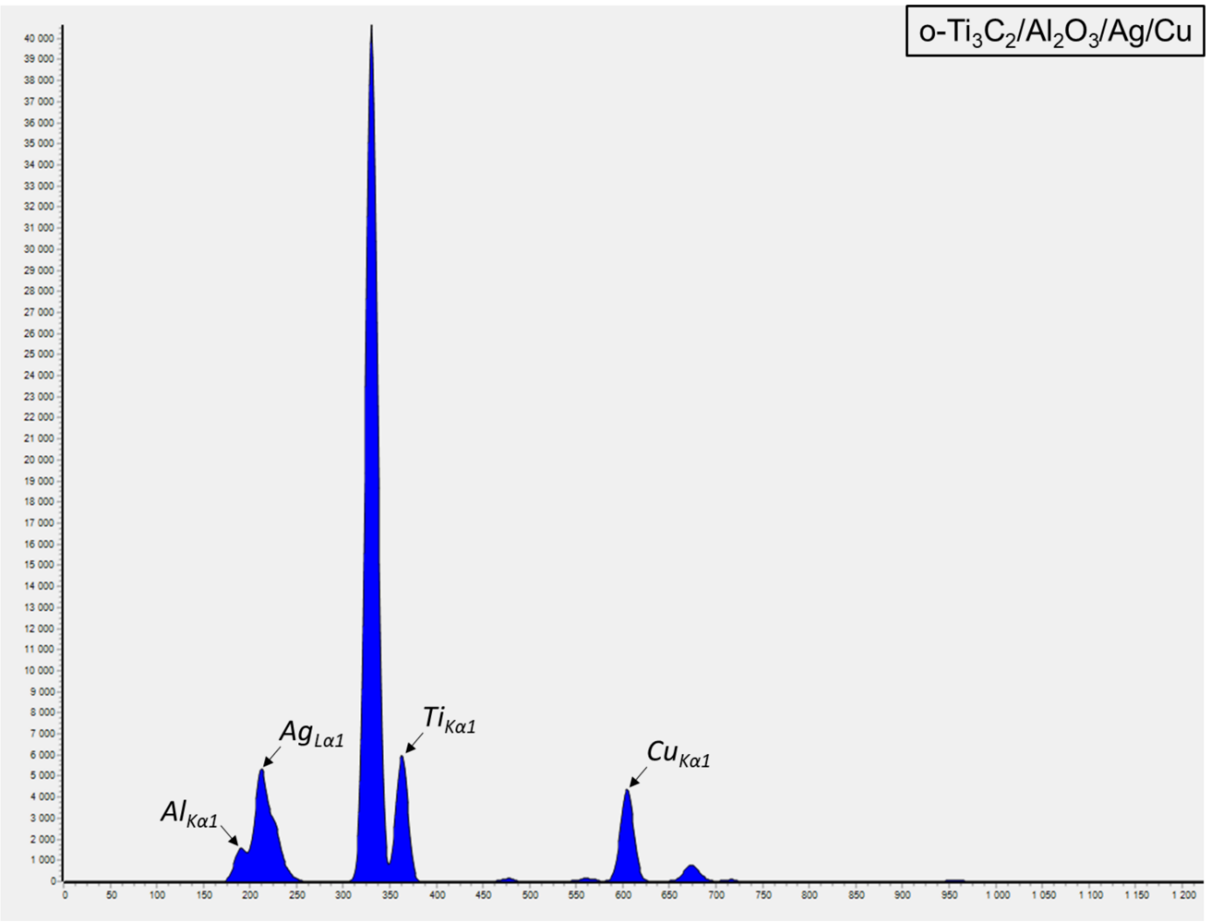

Figure S4. XRF spectra of o-Ti_3C_2/Al_2O_3/Ag/Cu-modified material.
